# Electronic cigarette liquids impair metabolic cooperation and alter proteomic profiles in V79 cells

**DOI:** 10.1186/s12931-022-02102-w

**Published:** 2022-07-15

**Authors:** Sara Trifunovic, Katarina Smiljanić, Albert Sickmann, Fiorella A. Solari, Stoimir Kolarevic, Aleksandra Divac Rankov, Mila Ljujic

**Affiliations:** 1 Biology of Robustness Group, Mediterranean Institute for Life Sciences, Split, Croatia; 2 Department of Biochemistry and Centre of Excellence for Molecular Food Sciences, University of Belgrade, Faculty of Chemistry, Studentski Trg 12-14, 11000 Belgrade, Serbia; 3 Leibniz-Institut Für Analytische Wissenschaften - ISAS - E.V., Bunsen-Kirchhoff-Straße 11, Dortmund, Germany; 4 Medizinische Fakultät, Medizinisches Proteom-Center (MPC), Ruhr-Universität Bochum, 44801 Bochum, Germany; 5 Department of Chemistry, College of Physical Sciences, University of Aberdeen, Aberdeen, AB243FX Scotland, UK; 6 Department of Hydroecology and Water Protection, Institute for Biological Research “Sinisa Stankovic”, National Institute of Republic of Serbia, University of Belgrade, Belgrade, Serbia; 7 Institute of Molecular Genetics and Genetic Engineering, University of Belgrade, Belgrade, Serbia

**Keywords:** Electronic cigarettes, Proteomics, Lung fibroblast, Metabolic cooperation

## Abstract

**Background:**

Although still considered a safer alternative to classical cigarettes, growing body of work points to harmful effects of electronic cigarettes (e-cigarettes) affecting a range of cellular processes. The biological effect of e-cigarettes needs to be investigated in more detail considering their widespread use.

**Methods:**

In this study, we treated V79 lung fibroblasts with sub-cytotoxic concentration of e-cigarette liquids, with and without nicotine. Mutagenicity was evaluated by HPRT assay, genotoxicity by comet assay and the effect on cellular communication by metabolic cooperation assay. Additionally, comprehensive proteome analysis was performed via high resolution, parallel accumulation serial fragmentation-PASEF mass spectrometry.

**Results:**

E-cigarette liquid concentration used in this study showed no mutagenic or genotoxic effect, however it negatively impacted metabolic cooperation between V79 cells. Both e-cigarette liquids induced significant depletion in total number of proteins and impairment of mitochondrial function in treated cells. The focal adhesion proteins were upregulated, which is in accordance with the results of metabolic cooperation assay. Increased presence of posttranslational modifications (PTMs), including carbonylation and direct oxidative modifications, was observed. Data are available via ProteomeXchange with identifier PXD032071.

**Conclusions:**

Our study revealed impairment of metabolic cooperation as well as significant proteome and PTMs alterations in V79 cells treated with e-cigarette liquid warranting future studies on e-cigarettes health impact.

**Supplementary Information:**

The online version contains supplementary material available at 10.1186/s12931-022-02102-w.

## Background

Electronic cigarettes (e-cigarettes) are a form of electronic nicotine delivery system that aerosolize the liquid typically containing propylene glycol, glycerol and flavouring agents, with or without nicotine, into aerosol. Use of e-cigarettes has gained significant popularity in recent years, especially among young adults and first time users of cigarettes, but also as a smoking cessation aid [1].

E-cigarettes are marketed as a safer alternative to classical cigarettes and several studies found that switch from classical cigarettes to e-cigarettes leads to improvement in health outcomes for long term tobacco users with chronic obstructive pulmonary disease (COPD) [2, 3], cardiovascular diseases [4, 5] and asthma [6]. Nicotine levels delivered by e-cigarettes are similar to those delivered by conventional cigarettes [7], however amounts of several toxicants and carcinogens were found to be lower in e-cigarette liquid and aerosols than in conventional cigarettes [8]. Still, the aerosol from e-cigarettes was found to contain harmful constituents such as acrolein, acetaldehyde, and formaldehyde and flavour components such as lycidol, acetol and diacetyl [9–11]. While some studies reported lower cytotoxicity and mutagenicity of e-cigarettes when tested in vitro [12–14], results demonstrating similar toxicity to cigarette smoke were also reported [15]. With the research on e-cigarettes constantly expanding, an increasing body of work points to their adverse effects, with the full extent of their biological consequences and mechanism of action still largely unknown.

Inflammation, oxidative stress, DNA damage and cell death are only some of the effects associated with e-cigarette liquids and aerosols exposure [16–20]. Exposure to e-cigarettes leads to changes in gene and miRNA expression as well as in DNA methylation patterns [21–26]. Given the relative novelty of e-cigarettes, clinical data on their long-term systemic health effects are still unavailable. Results of several comparative analyses between e-cigarettes users and non-smokers found that e-cigarette users have an increase in levels of oxidative stress and activation of pro-inflammatory lymphocytes and monocytes involved in inflammatory atherosclerosis, mitochondrial gene deregulation as well as deregulation of genes belonging to cancer functional network [25–27]. Carcinogenic and tumour promoting properties of e-cigarettes were also reported. E-cigarette exposure acted as a lung carcinogen and a potential bladder carcinogen in mice and mouse xenografts from patient-derived brain tumour treated with e-cigarette liquid had accelerated growth and worse prognosis compared to the control group [28, 29]. Exposure to e-cigarettes was found to increase cisplatin resistance in oral cancer cells, indicating their role in therapy-resistance in cancer as well [30]. E-cigarettes have also emerged as a risk factor leading to increased susceptibility to respiratory infections, including severe acute respiratory syndrome coronavirus 2 (SARS-CoV-2) [31–33].

Rising prevalence of e-cigarettes use, especially among young people, requires better understanding of their overall health effects. Characterizing the biological effects following the e-cigarette exposure is vital in evaluating the extent of their effect and estimating pathological consequences associated with their use.

This study was designed to investigate the effect of e-cigarette liquids with or without nicotine on V79 lung fibroblasts by assessing their mutagenic capacity, genotoxicity and metabolic cooperation ability followed by in-depth proteomics via high resolution analysis, using parallel accumulation serial fragmentation (PASEF), which compared protein expression profiles and provided relative profiling of chemical and posttranslational modifications across different proteomes. Our results indicate that e-cigarette liquids affect metabolic cooperation in V79 cells and lead to proteomic changes affecting global protein synthesis, mitochondrial function and focal adhesion.

## Methods

### Cells

Chinese hamster lung fibroblast V79 cells were purchased from American Type Culture Collection (ATCC). Cells were cultured in Dulbecco's Modified Eagle Medium (Sigma) supplemented with 10% (v/v) Fetal Bovine Serum (Gibco) at 37 °C in a humidified 5% CO_2_ atmosphere.

### Electronic cigarettes and nicotine treatments

Commercially available e-cigarette liquids (Virginia Tobacco flavoured) were purchased at the local store in Serbia. They were labelled to contain propylene glycol, glycerol and aroma, with or without nicotine (18 mg/mL) (termed ECL-N and ECL respectively). Nicotine solution (Sigma) was used as a reference treatment at the same concentration as the nicotine in ECL-N dilutions. For the initial screening on V79 cells, the e-cig liquids were used in dilutions of 0.69, 1.38, 2.77 and 4.14% (v/v) in V79 culture medium which in ECL-N liquid corresponded to nicotine concentrations of 125, 250, 500 and 750 µg/mL, respectively. All the following experiments in V79 cells were performed using e-cigarette liquids dilution of 1.38% corresponding to nicotine concentration of 250 µg/mL.

### MTT viability assay

Cells were seeded in 96 well plate at 5 × 10^3^ cells per well, and treated 24 h after seeding. After 72 h of treatments, Thiazolyl Blue Tetrazolium Blue (MTT) (Sigma) was added and incubated at 37 °C, 5% CO_2_ for 2 h. MTT formazan crystals were dissolved in dimethyl sulfoxide (DMSO) and absorbance was measured at 570 nm. Samples were normalized to untreated control.

### HPRT mutation assay

The HPRT (hypoxanthine–guanine-phosphoribosyl-transferase) assay was performed with modifications of a previously published method [34]. Using this assay, cells with loss-of-function HPRT mutations occurring after the treatments can be selected using 6-thioguanine (6-TG), as HPRT mutant cells are resistant to 6-TG and their number thus serve as a measure of mutagenic potential of a tested substance [35]. Briefly, 0.2 × 10^6^ cells were seeded in 150 mm Petri dish, treatments were added 24 h after seeding and cells were treated for 72 h. Treatment with 2 mM H_2_O_2_ for 1 h was used as a positive control. After the treatments, the survival and colony forming ability of V79 cells were determined by seeding 100 cells in 60 mm Petri dish (2 dishes per treatment). Cells were cultivated for 8 days and colonies were stained with crystal violet and counted. For HPRT mutagenesis, treated cells were seeded at 0.2 × 10^6^ in 150 mm Petri dish and cultivated for 6 days in drug-free media in order to use the entire remaining endogenous HPRT enzyme in HPRT mutant cells. After the recovery period, 0.25 × 10^6^ V79 cells were seeded in 15 cm Petri dishes (4 dishes per treatment) and cultivated in the presence of 7.5 µg/mL 6-TG (Sigma) for 8 days. The 6-TG resistant (6-TG-R) colonies were counted, normalized to the colony forming ability of the corresponding treatments and compared to untreated cells.

### Comet assay

Cells were seeded in 150 mm Petri dishes at 0.2 × 10^6^ cells per dish, and treated 24 h after seeding. After 72 h of treatments, cells were detached and suspensions were adjusted to 0.5 × 10^5^ cells per mL.

Comet assay was performed in standard format as described previously [36] with modifications. Microscope slides were precoated with 1% normal melting point agarose. Cell suspensions prepared as described above (30 μL) were mixed with 1% low melting point agarose. From that mixture, 70 µL was placed on precoated slides and left for 5 min at 4 °C for gels to solidify. Afterwards, slides were lysed for 1 h in freshly prepared, cooled (4 °C) lysis buffer (2.5 M NaCl, 100 mM EDTA, 10 mM Tris, 1.5% Triton X-100, pH 10) and then transferred to electrophoresis chamber containing cold (4 °C) alkaline electrophoresis buffer (300 mM NaOH, 1 mM EDTA, pH 13). The first step, denaturation of the DNA, involved the incubation of slides for 20 min at 4 °C without electricity, after which the current was released (0.75 V/cm, 300 mA) in the same solution for 20 min. After electrophoresis, the slides were transferred to a freshly cold (4 °C) neutralizing buffer (0.4 M Tris, pH 7.5) for 15 min. The slides were then fixed in methanol at 4 °C for 15 min and air dried at room temperature. For visualization, the gels were stained with GelGreen (Biotium). A total of 50 nuclei were analysed per slide (150 per treatment group) using fluorescent microscope under magnification 400 × , excitation filter 510–560 nm, barrier filter 590 nm (Leica, DMLS, Austria) and the Comet Score 2.0 software. Tail DNA % was used for quantification of the damage.

### Metabolic cooperation assay

Metabolic cooperation assay was conducted according to the previously published method with modifications [37]. The V79 cells were cultivated in the presence of 7.5 µg/mL 6-TG and colonies from spontaneously occurring resistant cells were selected and propagated to obtain 6-TG-R V79 cells.

Mix of 0.75 × 10^6^ WT and 100 6-TG-R cells were seeded in co-culture in 60 mm Petri Dish and treatments were added 4 h after seeding. As a control of the impact of each treatment on the colony forming ability of 6-TG-R cells, 100 cells were seeded separately in 60 mm Petri dishes and treated the same way as the co-culture Petri dishes. After 72 h, treatments were removed and fresh medium supplemented with 7.5 µg/mL 6-TG. Eight days after the seeding, Petri dishes were stained with crystal violet and 6-TG-R colonies were counted. The number of colonies in mixed Petri dishes was normalized to seeding controls with only 100 6-TG-R cells.

### Immunoblotting

Cells were washed with phosphate buffer saline (PBS), scraped from the Petri dish surface and lysed in RIPA buffer (Thermo Fisher) with Halt protease inhibitors (Thermo Scientific) for 90 min on ice. Undissolved cellular debris was removed by centrifugation at 13,000 rpm for 30 min at + 4 °C. Protein concentration was determined by BCA Protein Assay Kit (Thermo Fisher Scientific). Seven micrograms of proteins were loaded on 12% linear homemade gels (12% Acrylamide/bisacrylamide (Fisher Bioreagent), APS (Biosolve), TEMED (Sigma) and Tris–glycine buffer) and separated by gel electrophoresis using the Mini-Protean® Tetra Cell system (Bio-Rad). Proteins were then transferred to PVDF membrane using Trans-Blot Turbo Transfer System (BioRad) with 25 V constant (up to 1.0 A) for 30 min. Membranes were stained with MemCode Staining kit (Thermofisher) and blocked in PBS-Tween 0.05%-BSA 5% overnight at + 4 °C. Membranes were then incubated with Connexin 43 (Cx43) Monoclonal Antibody (Thermo Fisher), 1:500 at + 4 °C ON, followed with Anti-Tubulin antibody (Abcam) 1:100,000 at + 4 °C ON and goat anti-mouse Alexa Fluor 488 (Thermo Fisher) 1:5000 3 h RT and fluorescence was measured by Typhoon™ FLA 9500 biomolecular imager (GE Healthcare). Quantification of the signal was performed using ImageJ (National Institute of Mental Health; Bethesda, MA, USA) software. Cx43 expression was normalized to tubulin expression in each sample.

### V79 extracts preparation for Nano Liquid Chromatography coupled to tandem mass spectrometry (nLC-MS/MS)

For the shotgun in-gel digestion, cells were lysed with RIPA buffer and diluted extracts (30 μg or proteins per well) were resolved in reducing, one dimension, 12% sodium dodecyl sulphate–polyacrylamide gel (1D SDS-PAGE), only to a point to enter the separating gel up to 5 mm path. Proteomes in excised gel bands were reduced by 10 mM dithiothreitol and alkylated with 55 mM iodoacetamide and digested with proteomics-grade trypsin (Sigma-Aldrich, Taufkirchen, Germany) in a 1:30 enzyme to substrate ratio, overnight at 37 ℃ [38]. Trypsin digests were cleaned and desalted with HyperSep tips C18 (Thermo Scientific, Rockwood, TN, USA), evaporated and reconstituted in 30 μL of 0.1% trifluoro-acetic acid (TFA).

### nLC-MS/MS

Tryptic peptides, 2 μg from each samples (15 µL injection volume) were chromatographically separated with U3000 RSLC-nano online (Thermo Scientific, Bremen, Germany), coupled to a TimsTOF mass spectrometer (Bruker, Bremen, Germany). Peptides samples were loaded onto the trap column (Acclaim PepMap100 C18; 100 µm × 2 cm) in 0.1% TFA at a flow rate of 20 µL/min. After 5 min, the pre-column was switched in line with the main column (Acclaim PepMap100 C18; 75 μm × 50 cm) and peptides were separated using a 130 min binary gradient ranging from 3 to 38% acetonitrile in 0.1% formic acid at 60 °C and a flow rate of 250 nL/min. The mass spectrometer was operated in parallel accumulation serial fragmentation—data dependent acquisition (PASEF-DDA) mode, with duty cycle at 100%, accumulation time of 1.1 s and 10 PASEF MS/MS scans per top-N acquisition cycle. MS and MS/MS spectra were recorded from m/z 100 to 1700. A polygon filter was applied to the m/z and ion mobility plane to select features most likely representing peptide precursors rather than singly charged background ions. The quadrupole isolation width was set to 2 Th for m/z < 700 and 3 Th for m/z > 700, and the collision energy was ramped as a function of increasing ion mobility: 0.6 to 1.6 1/K0[V-s/cm3] – 20 to 59%.

### Identification and label free quantification of V79 cell treatments and their post-translational modifications profiling

V79 proteins were identified using the PEAKS X Pro platform (Bioinformatics Solution Inc., Ontario, Canada) and its PTM algorithm against a UniProtKB database (http://www.uniprot.org/) of *Cricetulus griseus* species (taxon ID 10,029, 56,575 sequences, accessed 22/08/2021) and contamination database as common Repository of Adventitious Protein entries (http://www.thegpm.org/, 116 sequences, accessed 18/10/2019). The list of all identified V79 proteins (Additional file 8: Table S1) consists of protein groups, with at least two unique peptides, contaminant hits filtered out and protein false discovery rate (FDR) less than 0.5%.

The PEAKS X Pro platform included 313 post-translational and chemical modifications in the search space regularly updated from the Unimod web-based database. The quantity of identified V79 proteins was compared by label free quantification (LFQ) using the PEAKS Q algorithm. The term “PTM profiling” means relative extent of the modifications within a single sample. Relative profiling of PTMs and label free relative quantification of proteins in 4 groups with one sample per group was done as described previously [39]. Briefly, in LFQ section, cut off filter criteria for defining confident differently regulated proteins were significance score of 20 (−10logP, p < 0.01), twofold change in log2 up/down ratio and at least one used peptide, affecting any of the 3 treatments in respect to the control group.

The bioinformatics, with functional enrichment analyses and Venn diagrams were done in Functional Enrichment Analysis Tool—FunRich software v 3.1.3 (http://funrich.org) and QuickGO (https://www.ebi.ac.uk/QuickGO). As background database, a complete UniProt set of *Cricetulus griseus* species (taxon ID 10,029, 56,575 sequences) was used, unless otherwise stated. The mass spectrometry proteomics data have been deposited to the ProteomeXchange Consortium (http://proteomecentral.proteomexchange.org) via the PRIDE [40] partner repository with the dataset identifier PXD032071 and 10.6019/PXD032071.

### Statistics

The statistical analysis of the results was performed using GraphPad Prism version 8.4.3 (La Jolla, CA, USA) or Statistica 6.0 Software (StatSoft, Inc.). Normality was tested by Shapiro–Wilk test and one-way ANOVA followed by Dunnett’s multiple comparisons test or Kruskal–Wallis one-way ANOVA were applied. Differences with a p-value lower than 0.05 were considered statistically significant.

PEAKS Q statistical test was used within PEAKS Studio proteomic platform to test for significant differences in relative label free protein quantification. Hypergeometric test as an uncorrected p value assessment method, and Bonferroni test, together with BH and Q-value (Storey-Tibshirani method) tests as corrected versions of p-value assessments were used by FunRich software version 3.1.3.

## Results

### Assessment of mutagenic capacity (HPRT assay) and genotoxicity (comet assay) of e-cigarette liquids

**Fig. 1:**
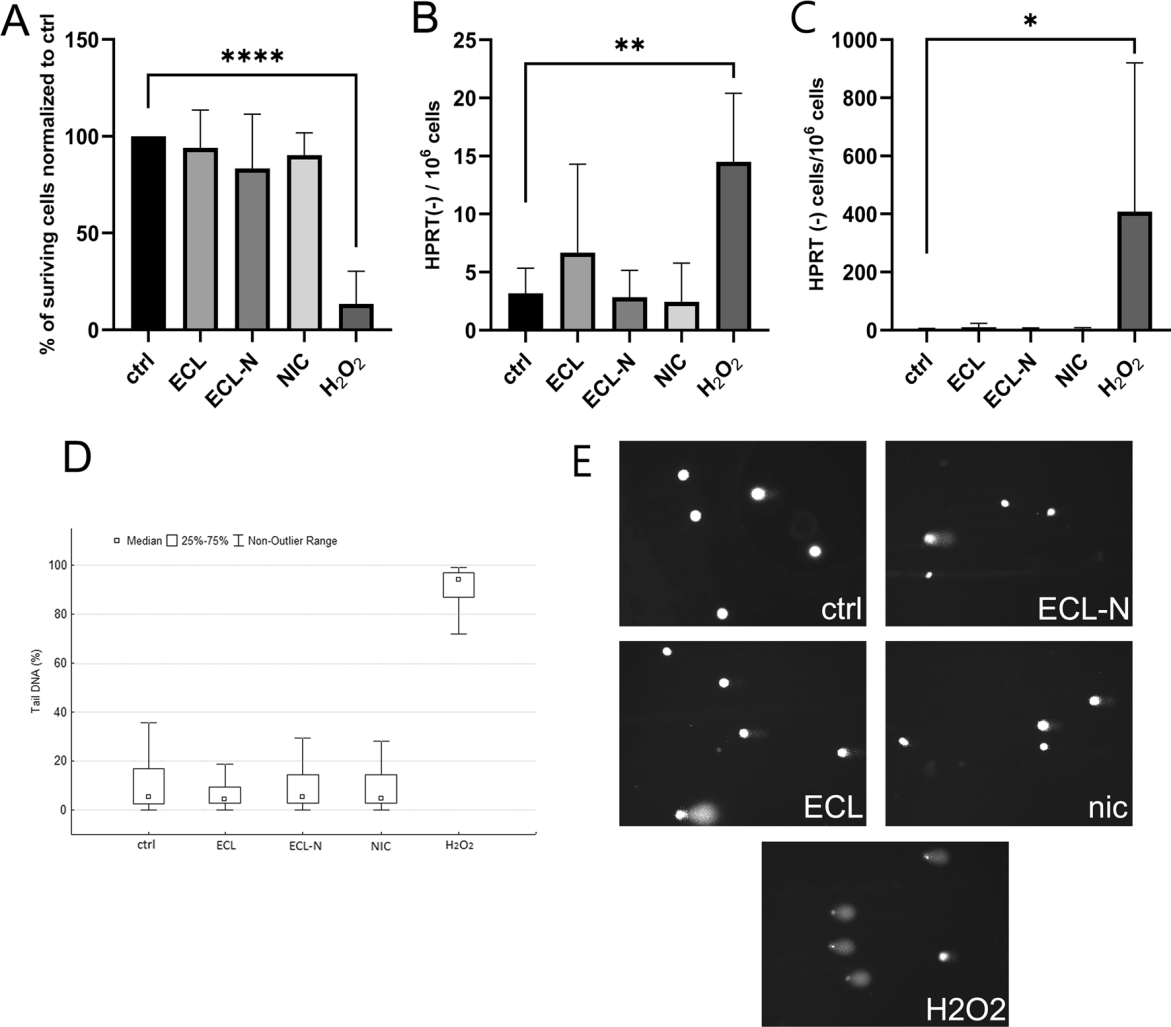
Mutagenicity and genotoxicity of e-cigarette liquids in V79 cells. **A** Colony forming ability of V79 cells after 72 h (ECL; ECL-N; NIC) and 1 h (H_2_O_2_), treatments normalised to control (ctrl). **B** Number of HPRT(-) cells per 1 × 10^6^ seeded V79 cells after 6 days of recovery from treatments. **C** Number of HPRT(-) cells per 1 × 10^6^ cells normalised to colony forming ability after treatments. **A**–**C** Data are presented as mean ± SEM from six independent replicates. **D** The level of DNA damage in V79 cells determined by single cell gel electrophoresis (Comet assay)—tail DNA (%) of V79 cells after 72 h (ECL; ECL-N; NIC) and 1 h (H_2_O_2_) treatments. Data are representative of three independent experiments. **E** Representative images of tail DNA (comets) of treated V79 cells

For all the in vitro assays, we used e-cigarette dilutions of 1.38%, equivalent to 250 µg/mL of nicotine in ECL-N, which was found to be non-cytotoxic by MTT assay (Additional file 1: Figure S1). We found no increase in number of HPRT deficient cells after e-cigarette liquid and nicotine treatments, indicating that tested e-cigarette liquids, at the concentrations used in this assay, were not mutagenic in V79 cells (Fig. 1A–C). H_2_O_2_, previously described as mutagenic in V79 cells, was used as a positive control in this assay. Additionally, genotoxicity was tested by comet assay, results of which were in accordance with results of HPRT assay as it showed no significant genotoxic potential of e-cigarettes (Fig. 1D, E).

### Effect of e-cigarette liquids on metabolic cooperation in V79 cells

This assay is based on co-culturing HPRT deficient cells with wild type (metabolically proficient) cells which transfer toxic 6-TG metabolites to HPRT deficient cells through intercellular communication thus rendering them sensitive to 6-TG. Chemicals reducing cell communication through gap junctions would therefore lead to increase in number of 6-TG resistant colonies.

**Fig. 2:**
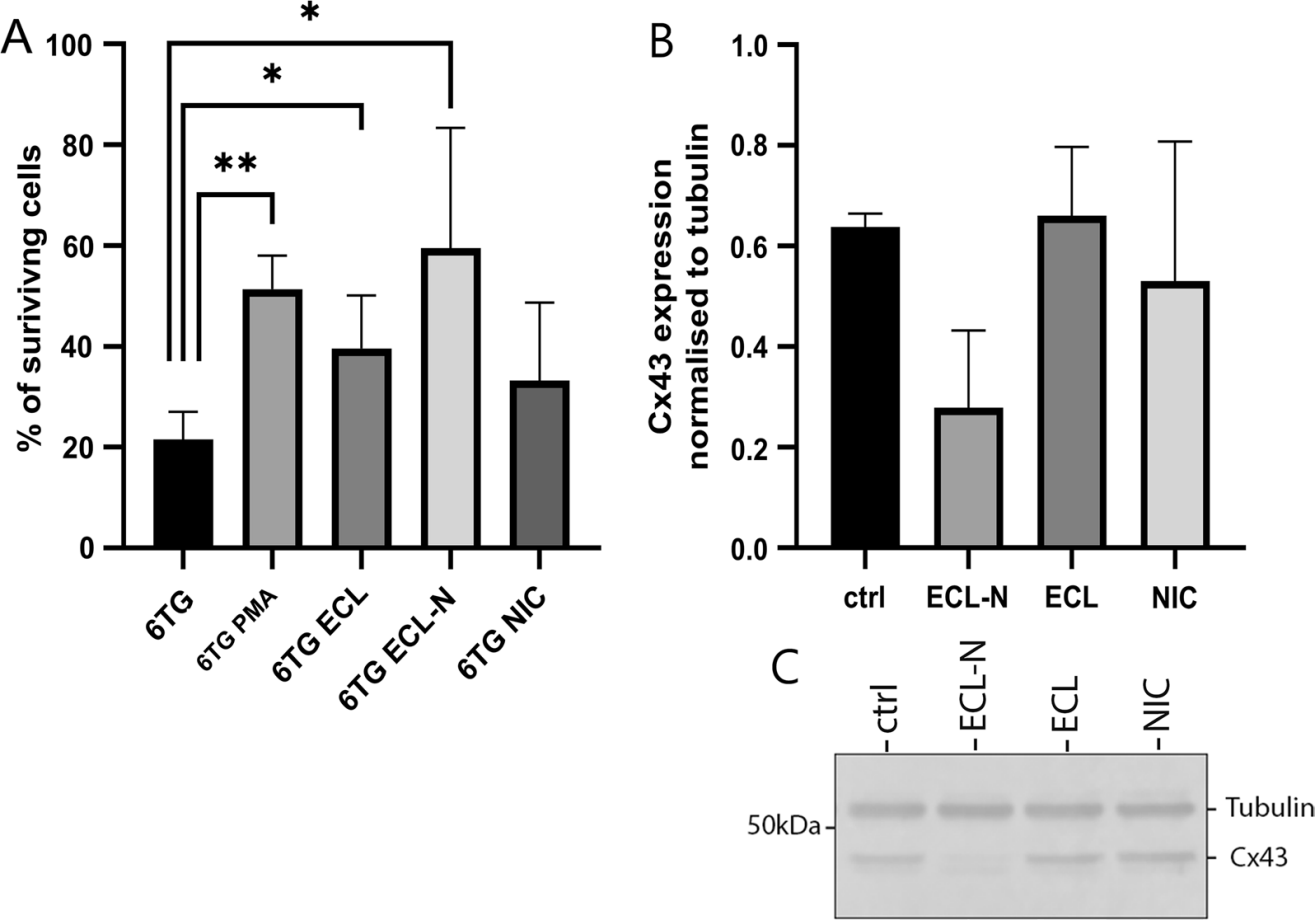
Effects of e-cigarette liquids on metabolic cooperation and Cx43 protein expression. **A** Metabolic cooperation: number of 6-TG-R V79 surviving colonies in mixed co-cultures of 100 6-TG-R and 0.75 × 10^6^ WT cells normalised to seeding controls (100 6-TG-R cells treated the same way as co-cultures). **B** Densitometric quantification of Cx43 protein expression normalised to tubulin. Data are presented as mean ± SEM from three separate experiments of independent cell preparations. * p < 0.05; **p < 0.01 versus negative control. **C** Western blot of Cx43 and tubulin as endogenous control in V79 cells after 72 h treatments, blot is representative of three separate experiments and cropped from original image (Additional file 7: Fig. S7)

Using metabolic cooperation assay we found that treatments with e-cigarette liquids, both ECL and ECL-N, significantly increased number of 6-TG resistant colonies in V79 cells (39.59 ± 4.71% and 59.51 ± 10.67% respectively vs. 21.52 ± 2.4% for control; p < 0.01), indicating impaired intercellular communication between wild type and HPRT deficient cells (Fig. 2A). Phorbol 12-myristate 13-acetate (PMA) was used as a positive control. Based on the results of mutagenic HPRT assay (Fig. 1), the increase in number of 6-TG resistant colonies cannot be attributed to the emergence of new HPRT deficient cells following treatments and is due to disruption of metabolic cooperation.

### Effect of e-cigarette liquids on Cx43 protein expression

We next analysed the expression of Cx43 in V79 cells using western blot and observed lower Cx43 expression in ECL-N treatment (44 ± 25.3%), however there was no statistical difference for none of the treatments (103 ± 17.9% ECL and 82.1 ± 39.7% NIC) (Fig. 2B).

### V79 proteome coverages and LFQ of differently regulated proteins

The proteomic profiles of V79 cells induced by e-cigarette liquids were investigated using TimsTOF shotgun proteomics. According to the human protein atlas data on the number of expressed proteins in lung tissue of 14,490 (https://www.proteinatlas.org/humanproteome/tissue/lung), and total number of genes in *Cricetulus griseus* species of 15,308 [41], a rough approximation of the maximum possible number of expressed proteins within V79 fibroblast proteome can be drawn. In this context, we have covered at least one third of V79 theoretical proteome maximum by our TimsTOF, DDA-PASEF shotgun approach (Additional file 2: Figure S2, Additional file 8: Table S1), similar to the study of Liu et al., examining the inhibition of V79 fibroblast proliferation in the deep underground environment [42].

**Fig. 3:**
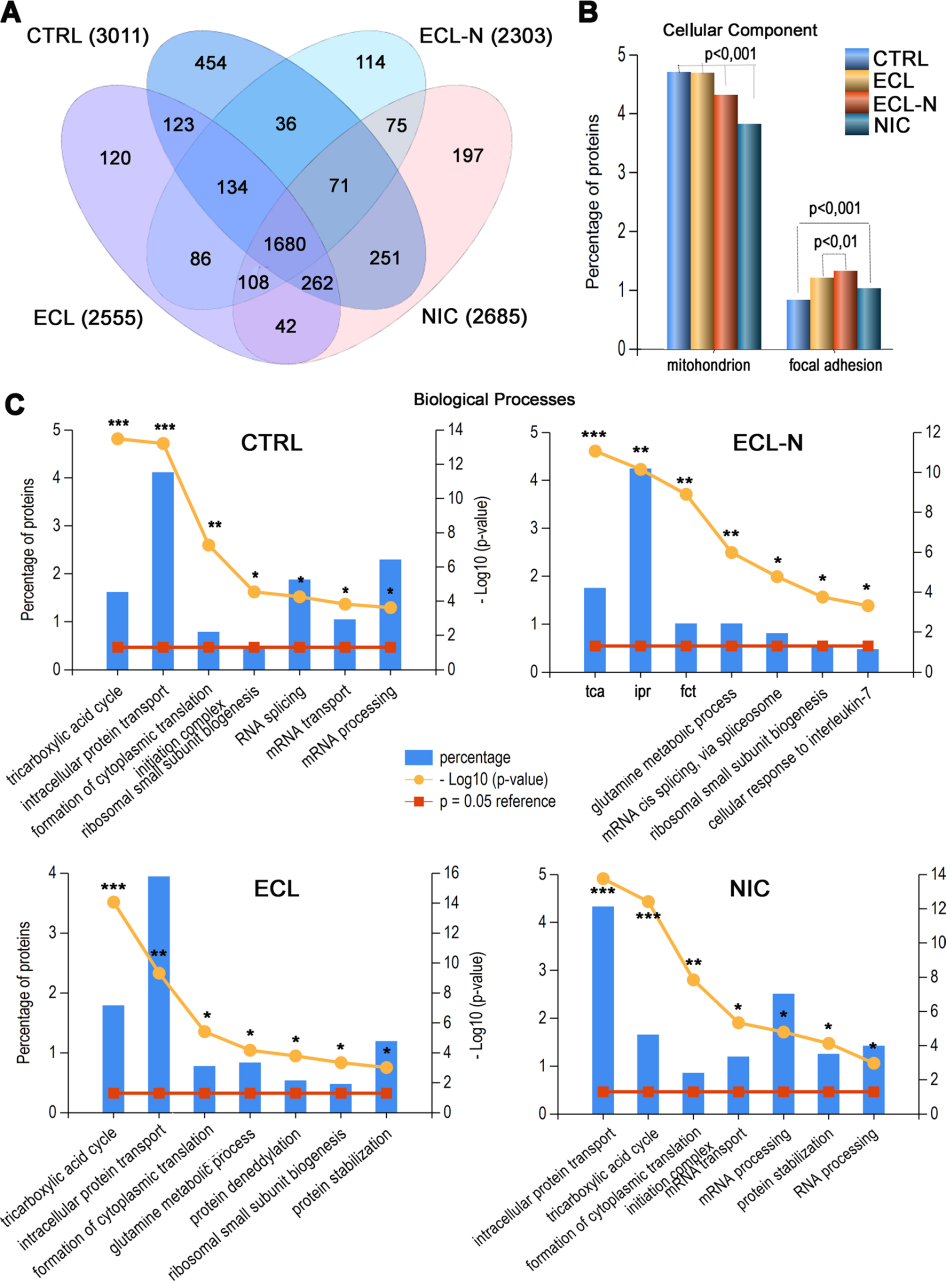
Proteomes overlap of V79 cell treatments and their bioinformatics profiling in respect to biological processes. **A** Venn diagram of V79 treatments top proteins (e.g. in case when several proteoforms with different accession entries are present, only the top scored are reported); the numbers in the brackets denote total top proteins in each treatment. **B** Cellular component comparisons among V79 treatments with statistically significant enrichment of gene products in representative examples of cell compartments; p values displayed were calculated by hypergeometric test. **C** Biological processes profiling and its enrichment analysis in V79 lung fibroblast treatments. Legend: -log10 (p-value), the probability score in the gene ontology enrichment analysis; tca, tricarboxylic acid cycle; ipr, intracellular protein transport; fct, formation of cytoplasm translation initiation complex. All graphics were done in FunRich 3.1.3 software

Overall, we found that the untreated cells had the highest number of different proteins, approximately 10% more than pure nicotine treatment, 15% more than ECL and 23% more than ECL-N, indicating the most intensive shutdown of protein products in ECL-N treatment (Fig. 3A). The number of uniquely identified proteins in each treatment followed the same pattern as the total number of proteins identified, with highest number in control and lowest in ECL-N treatment (Fig. 3A). This result is more likely a consequence of treatments than of changes in total ion current chromatographic profiles that show high reproducibility (Additional file 3: Figure S3).

**Fig. 4:**
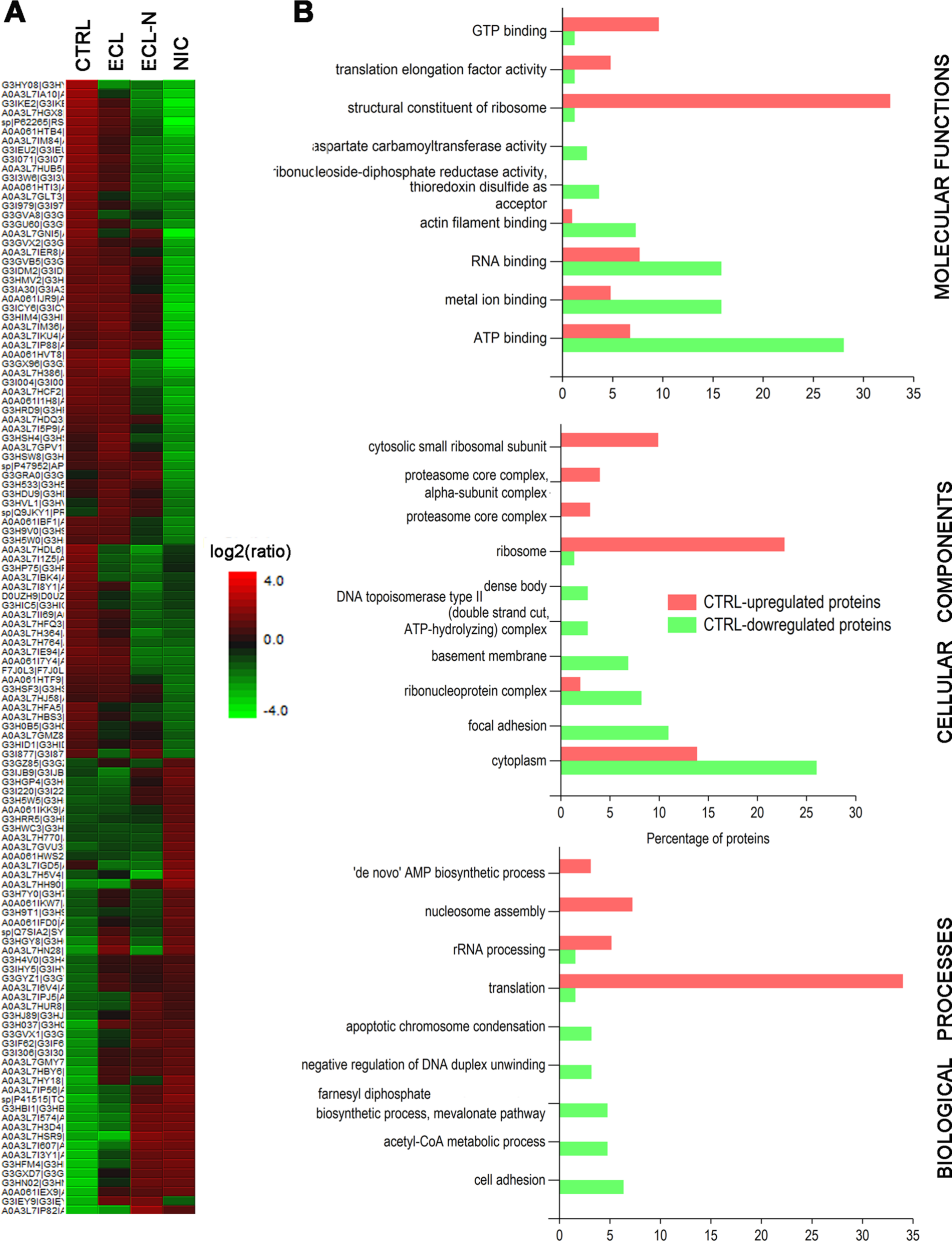
Relative proteomic quantification of differently regulated proteins in V79 cell treatments and their bioinformatics comparison. **A** Heat map of relative, label-free protein quantification (LFQ) of V79 cell line treatments displaying the protein groups that passed the filters (only significantly up- and downregulated proteins in respect to control, at p < 0.01 done with PEAKS Q statistical test). The hierarchical clustering was generated using neighbour-joining algorithm with a Euclidean distance similarity measurement of the log2 ratios of the protein abundance of each group relative to the average abundance. **B** Gene ontologies with significant enrichment (p < 0.05, according to a hypergeometric and Bonferroni tests) in control-upregulated (red bars) and control-downregulated (green bars) proteins, in respect to molecular function, cellular component and biological processes aspects done in FunRich 3.1.3 program

The heatmap of significantly upregulated and downregulated proteins by LFQ approach revealed that the ECL heatmap pattern of differently regulated proteins resembled heatmap of untreated cells, while the pattern of ECL-N resembled more the NIC treatment heatmap pattern (Fig. 4A). There are no proteins in ECL or ECL-N treatment that are uniquely down- or upregulated (solely differently regulated in either ECL or ECL-N). The greatest difference exists between the control and NIC treatment to that extent that it stands what is upregulated in the control is downregulated in NIC treatment and vice versa (Fig. 4A, B). The list of confidently differently regulated proteins (ratio of log2 ≥ 2) and proteins that were potentially differently regulated (ratios of log2 between 1.3 and 2) as revealed by relative label-free quantification is presented in Additional file 9: Table S2 that was sorted against ECL-N log2 ratio in descending order, showing firstly the most upregulated proteins.

When examining the cell compartmental aspect of all top proteins identified in different treatments, mitochondrion and focal adhesion were prominent cell components with significant enrichment and substantial differences between treatments and control (Fig. 3B). Other significant and less occupied compartments included proteasome core complex and cytosolic stress granules (Additional file 10: Table S3). Mitochondrial proteins were depleted by 20% in nicotine treatment compared to the control, while all three treated V79 cell groups were more abundant with proteins involved in focal adhesion, with ECL-N treatment being 60% higher than control (Fig. 3B). A closer look at the constituents of mitochondrial compartment revealed 95, 81, 68 and 70 protein species in untreated, ECL, ECL-N and NIC cell groups, respectively, with 18 truly unique and essential protein products found only in untreated cells, such as glutaminase (UniProtKB ID—Q7TQN0) and dihydrofolate reductase (UniProtKB ID—Q2MH30) among others (Additional file 11: Table S4). Dihydrolipoyl dehydrogenase, mitochondrial (UniProtKB ID—Q8CIZ7) was exclusively found in ECL-N treatment and in various cell projections—nucleus and cytosol vesicles, while vacuolar protein sorting-associated protein 35 (VPS35) (https://www.uniprot.org/uniprot/A0A3L7HZ98), was the unique entry in NIC treatment.

In-depth examination of the qualitative differences of focal adhesion constituents revealed that the untreated cells had the lowest number of proteins [17] with a single unique entry of 59 kDa serine/threonine-protein kinase (UniProtKB ID—C0SSX9) which is an integrin-linked protein kinase involved in positive regulation of substrate adhesion-dependent cell spreading and fibroblast migration. In contrast, e-cigarette and nicotine treatments had three isoforms of non-specific protein-tyrosine kinase (UniProtKB IDs—A0A061IJK6, A0A061ILL2 and G3I7E2), that upon blast and alignment analyses, revealed 95% sequence identity with focal adhesion kinase 1 (FAK-1) of mouse species (*Mus musculus*). Treatment with ECL-N had the most of unique proteins in focal adhesion component aspect, e.g. three entries that corresponded to LIM and senescent cell antigen-like-containing domain protein (UniProtKB IDs—A0A3L7ILY6 and G3HJ13) and Src homology 2 (SH2) domain-containing protein (UniProtKB IDs—G3H402). The Cx43 levels in ECL and ECL-N treatments determined by proteome analysis followed the similar pattern as in western blot analysis (Additional file 9: Table S2).

The overall pattern of the most abundant biological processes that are significantly enriched among treatments is given in Fig. 3C. The first bar in each graph of Fig. 3C represents the most significantly enriched process with concomitant descending order. Therefore, any change in the order of biological processes among treatments in respect to control, means substantial reorganization of V79 cell functioning. For example, a tricarboxylic acid cycle, a core catabolic process for the energy production happening in mitochondria, is on the second position in nicotine-treated cells, in contrast to all other groups where it fits first position (Fig. 3C). This result is in accordance with the decreased participation of protein products in mitochondrion in nicotine-treated cells (Fig. 3B). Cells treated with ECL and ECL-N show inclusion of glutamine metabolic process, indicative of increased demand for ATP, biosynthetic precursors, and/or reducing agents. This prominent aspect in biological processes in ECL and ECL-N is supported by their increased contribution to focal adhesion assembly (Fig. 3B), pointing to increased response to stimuli or increased motility. Another specificity of ECL treatment includes appearance of protein deneddylation, while deneddylation executed by COP9 signalosome is significantly depleted in the ECL-N and NIC treatments. Additionally, response to interleukin-7 processes also appeared only in ECL-N treatment (Fig. 3C).

When comparing involvement of unique proteins from different treatments in biological processes, we could observe that unique proteins in untreated cells had sole participation in tRNA processing, response to interferon beta, positive regulation of endothelial cell proliferation and positive regulation of growth (Additional file 3: Figure S3). In addition, unique participation in negative regulation of actin filament depolymerisation and lipid transport are visible in ECL uniquely identified proteins. In more details, cholesterol metabolism and its transport are prominent hallmarks of ECL treatment (Additional file 3: Figure S3).

Cell components (CC), molecular functions (MF) and biological processes (BP) occupied by upregulated and downregulated proteins of the control are depicted in Fig. 4B. The most prominent gene ontology feature in upregulated control proteins is translation aspect via depleted structural constituents of ribosomes, ribosomes and translation, as revealed from molecular functions, cell components and biological processes, respectively. In a closer look to molecular functions and biological processes comparisons of upregulated proteins in all treatments, the translation process decreases in following order: control: ECL:ECL-N:NIC (Additional file 4: Figure S4 and Additional file 5: Figure S5). Looking at the most prominent gene ontology features in control-downregulated protein set (NIC-upregulated proteins), ATP-, metal-ion-, and RNA-binding in MF and cytoplasm in CC emerge, while there are several categories in BP (mevalonate pathway, acetyl-CoA metabolic process and cell adhesion) that can account for differences observed, since these processes happen in a cytoplasm and require ATP (Fig. 4B). Most of the gene ontologies where NIC upregulated proteins are prominent, follow the decreasing order NIC:ECL-N:ECL:CTRL in MF (Additional file 4: Figure S4) as well as in BP aspect (Additional file 5: Figure S5).

### E-cigarette liquids increase abundance of post-translational and chemical protein modifications

The representative examples of post-translational and chemical modifications (PTMs) profiling, with the substantial differences in their relative abundances (ratio) and share (percentage of saturation within the proteome) are given in Table 1. The complete list of all modifications and specific peptides and proteins that were affected, is given in Additional file 12: Table S5, which represents proprietary PEAKS XPro PTM algorithm that generated PTM profiling.

**Table 1:** Relative modification profiling of carbonylation, oxidative and e-cigarettes biohazard chemical modifications among control and treated V79 cell proteomes

Modification type	Control		ECL		ECL-N		NIC	
	Ratio*	%**	Ratio	%	Ratio	%	Ratio	%
*I Carbonylation due to direct oxidation of Lys. Thr and Pro amino acid side chains*								
2-pyrrolidone from Pro	1	0.03	1.8	0.07	1.4	0.07	1	0.04
2-amino-3-oxo-butanoic_acid from Thr	1	0.15	0.04	0.001	0.02	0.004	0.01	0.002
Lysine oxidation to aminoadipic semialdehyde	N/A	0	∞	0.001	N/A	0	N/A	0
*II Carbonylation due to Michael addition reaction of α.β-unsaturated aldehydes derived from lipid peroxidation*								
Levuglandinyl-lysine anhyropyrrole adduct	1	0.01	2.5	0.05	4	0.11	0.7	0.02
4-hydroxynonenal (HNE)	N/A	0	∞	0.001	∞	0.003	N/A	0
*III Carbonylation events due to AGEs formation*								
Carboxymethyl on N term	N/A	0	∞	0.004	∞	0.002	N/A	0
*Other direct oxidative modifications*								
Met single oxidation (sulfoxide)	1	54	0.8	66	0.7	78	0.8	68
Met double oxidation (sulfone)	1	0.02	5.9	0.2	4.6	0.2	1.9	0.06
His and Trp oxidation	1	0.09	5.9	0.77	4.8	0.85	3.7	0.51
Proline oxidation to pyroglutamic acid	N/A	0	N/A	0	∞	0.003	N/A	0
Tryptophan oxidation to hydroxykynurenin	1	0.01	1.2	0.02	0.9	0.02	1.4	0.02
*Chemical modification events most likely caused or enhanced by E-cigarette liquid and nicotine*								
2.3-dihydro-2 2-dimethyl-7-benzofuranol N-methyl carbamate	N/A	0	N/A	0	∞	0.001	N/A	0
5-dimethylaminonaphthalene-1-sulfonyl	N/A	0	∞	0.004	∞	0.001	∞	0.002
Hydroxymethyl	N/A	0	∞	0.0003	N/A	0	N/A	0
O-Isopropylmethylphosphonylation	N/A	0	∞	0.003	N/A	0	N/A	0
Tri nitro benzene	N/A	0	∞	0.1	∞	0.1	N/A	0
Diethylation	N/A	0	∞	0.002	N/A	0	N/A	0
Acetaldehyde + 26	1	0.01	1.8	0.03	1.5	0.04	1.4	0.03
Dichlorination of tyrosine residues	1	0.0004	45	0.03	32	0.03	27	0.02
Ethylation	1	0.01	2.7	0.05	1.4	0.04	3.4	0.07
Replacement of 2 protons by nickel	1	0.08	1.3	0.16	1.1	0.18	1.1	0.14

*Ratio: sum of areas under extracted ion chromatography (XIC) curves of all modified peptides bearing the same modification type divided by the sum of XIC curve areas of modified peptides from the control group. ** Percentage (%): share of the sum of XIC curve areas of all modified peptides with certain modification type, within summed XIC areas of all proteins per treatment (e.g. share within the complete proteome of the particular treatment). N/A: not applicable since modified peptide form does not exist (e.g. only unmodified form of peptide is present). ∞: when dividing integer with 0 as in the case when expressing ratio where XIC value of control modified peptide is 0

For an easier comprehending of chemical types of changes that were observed in V79 control and treated cell proteomes, Table 1 is divided into 5 sections. They include three types of carbonylation events (direct oxidation of certain amino acid side chains; Michael addition reaction of α,β-unsaturated aldehydes derived from lipid peroxidation; AGEs formation), other direct oxidative modification and modification events most likely caused or enhanced by e-cigarette liquid and nicotine. The richest repertoire of modification is observed within ECL treatment with 19 out of 21 representative modification types present. Somewhat less is seen in ECL-N treatment (17/21), while control and NIC groups have 11 and 12 out of 21 modification types, respectively. All these 21 modification types presented in Table 1 are irreversible except single methionine (Met) oxidation to sulfoxide [43, 44].

Most of the PTMs observed (19/21) follow the same trend in both abundancy aspects (the ratio of modified peptides and its saturation within the proteome) as they are increased in ECL and ECL-N treatments compared to control and nicotine. This demonstrates increased extent of oxidative stress in treatments. The only two modifications that do not follow this trend are Met oxidation to sulfoxide and direct oxidation of threonine (Thr) to 2-amino-3-oxo-butanoic acid. Ratio of Met oxidation to sulfoxide is slightly lower in the treated V79 cells, however its saturation is 24% higher in the ECL-N treatment compared to the control, and this is not negligible. One of the most prominent PTM changes was the presence of 4-hydroxynonenal (4-HNE), found only in treatments with e-cigarette liquids.

Additionally, chemical imprints on various proteins of several organic compounds with aliphatic and aromatic nature considered as potentially threatening in regard to respiratory health were identified, such as acetaldehyde, ethylation, diethylation, hydroxymethyl, either being totally absent from control or present in traces, compared to treated V79 cells (Table 1 and Additional file 11: Table S4). We have confidently detected other organic biohazard modifications such as O-isopropylmethylphosphonylation (organophosphate pesticide) present only in ECL and tri nitro benzene, a highly toxic compound [45] found only in ECL and ECL-N treatments.

## Discussion

Despite being widely considered a safer alternative to conventional cigarettes, e-cigarettes were shown to exert biological effects with marked clinical implications and therefore pose a serious public health issue. So far, the majority of in vitro studies of e-cigarettes effects on transcriptome and proteome focused on epithelial cells [22, 46–49] and not many data are present on fibroblasts. Lung fibroblasts, among other functions, have an important role in maintaining structural integrity and extracellular matrix of the lung and changes in their phenotype are associated with pathological conditions such as fibrosis and cancer progression [50, 51]. The V79 cell line has been widely used in detection of possible genotoxicants [52] and we utilized this feature to evaluate the mutagenicity and genotoxicity of e-cigarette liquids and their effect on metabolic cooperation with an addition of comprehensive proteome analysis. To our best knowledge, this is the first report on e-cigarette effect on fibroblast proteome.

Mutagenic capacity of e-cigarette liquids was assessed using HPRT assay, one of the most widely used somatic cell mutation assays, which has not been used for testing of e-cigarette liquids so far. Additionally, genotoxicity was assessed by comet assay. Results of mutagenic and genotoxic assessment by indicated assays are in agreement with previous studies that found no significant mutagenicity/genotoxicity of electronic cigarettes [13, 14], including micronucleus assay in V79 cells [53].

On the other hand, we found that both liquids negatively affected metabolic cooperation as assessed by metabolic HPRT assay, indicating impaired intercellular communication between wild type and HPRT deficient cells. The metabolic cooperation assay has been historically used to study gap junctional intercellular communication as it requires cell-to-cell contact and has also shown a level of correlation with carcinogenesis [54–57]. Cigarette smoke condensate has been previously shown to disrupt metabolic cooperation in V79 cells [55], but so far there were no reports on using this assay for studying e-cigarettes. Biological consequences of impaired intercellular communication are broad and this effect of e-cigarettes remains to be studied in detail. In addition to exchange of different molecules, metabolic cooperation between cells provides a mechanism for maintaining normal cell and tissue functioning by compensating and rescuing mutant phenotypes and keeping the pathological condition in the state of latency [58, 59]. Disruption of this cooperation can lead to loss of coordinated cellular response in the tissue and potentially allows the onset of initiated pathologies.

Proteome profiling of V79 cell after e-cigarette treatments revealed that e-cigarette liquids as well as nicotine alone induced significant depletion in total number of proteins in V79 cells. The most prominent effect was observed in cells treated with ECL-N, suggesting synergistic effect of e-cigarette liquid and nicotine. In agreement with this, when analysing differences in biological processes, molecular functions and cell components aspects, the translation aspect was significantly downregulated in all three aspects. Our findings support the finding of transcriptomic analysis by Park et al. 2021, who determined the reduced expression of ribosome genes and reduction in protein biogenesis in primary normal human bronchial epithelial cells treated with e-cigarette [22]. In the context of our study, the treatment type of Park et al. 2021 study corresponds to our ECL-N treatment that exhibited three- to fourfold decrease of translation process compared to the control. Similarly, activation of unfolded protein response, which attenuates protein synthesis while increasing protein folding, transport and ER-associated protein degradation, has been reported in normal human oral keratinocytes [60].

Mitochondrial proteins were among the most significantly depleted cell components in e-cigarette liquid treatments. While essential proteins such as glutaminase and dihydrofolate reductase were absent from treated cells, VPS35, involved in mitochondrial fragmentation, was present in nicotine treatment and dihydrolipoyl dehydrogenase was found in ECL-N. Inclusion of glutamine metabolic process in biological processes in cells treated with e-cigarette liquids can be an adaptation to mitochondrial dysfunction [61] but is also a characteristic of many cancer types, including lung cancer [62]. Cigarette smoke has been previously found to affect glutamine metabolism both in vitro [63] and in vivo [64] and our results point to similar effects of e-cigarette liquids. Given the biological implications, this shift in the glutamine metabolism in V79 cells treated e-cigarette liquids should be further explored.

Metabolic shift to glutamine also indicates increased demand for ATP, as in the case of cells with increased motility, which is supported by the increased contribution to focal adhesion assembly in cells treated with e-cigarette liquids. Focal adhesions are large and dynamic protein structures that form a link between cell cytoskeleton and the extracellular matrix. They act as scaffolds for many signalling pathways and transmit mechanical tension generated within the cell to extracellular surrounding and contribute to cell migration [65, 66]. This feature was most prominent in cells treated with ECL-N. FAK-1, found in both e-cigarette liquid treatments, is an important mediator of various signalling networks and is frequently overexpressed in cancer, where it promotes important malignant features [67, 68]. Enhanced adhesive signalling, including activation of FAK-1, is a fibroblasts hallmark of lung fibrosis, and FAK-1 has been proposed to be a key mediator of fibrotic disease [69]. This increase in focal adhesion proteins in treated V79 cells compared to untreated cells could indicate a potentially higher migration capacity of cells exposed to ECL, ECL-N and NIC, that can be a mark of pulmonary fibrosis and tumour cell invasion. Higher cell motility is also associated with a decrease in gap junction communication [70], which could partly explain the observed reduction of metabolic cooperation in treated V79 cells. The e-cigarettes have been previously reported to reduce Cx43 expression in osteoblasts, while effects of nicotine on Cx43 in the lung were found to be cell specific [71–74]. Whether gap junctions are functionally impaired by e-cigarettes in V79 cells or if other forms of intercellular communication, such as tunnelling nanotubes (TNTs), are involved, remains to be examined. Cx43 has a vital role in gap junction communication but is also involved in formation of TNTs [75]. It has a role in different cellular processes including proliferation, immunity, and transcriptional regulation among others [76, 77] and its dysregulation is known to have a part in different lung pathologies [78, 79]. Together with the results of metabolic cooperation assay, these results indicate that e-cigarette liquids impair intercellular communication in V79 lung fibroblasts, extent of which needs to be further investigated.

We also observed significant changes occurring in PTMs after the treatments with e-cigarette liquids. These changes indicate increased oxidative stress conditions and could affect protein function and contribute to adverse health effects caused by e-cigarettes. 4-HNE, found only in cells treated with e-cigarette liquids, is a marker of oxidative stress and lipid peroxidation [80, 81]. 4-HNE is a highly reactive compound that can form adducts with cellular proteins and DNA, and can function as a signalling molecule, activating stress response mechanisms and modulating the activity of redox-sensitive transcription factors [82]. Increased levels of 4-HNE have been identified in several in vitro and in vivo studies after the exposure to e-cigarette vapour or cigarette smoke [83–85]. 4-HNE has been involved in pathophysiological changes of the non-malignant tissue in the vicinity of cancer [86, 87] and its increased levels have been linked to chronic obstructive pulmonary disease [88]. Reduction of 4-HNE levels has been linked with clinical improvement in patients with pulmonary arterial hypertension [89] and improvement in oxidative stress after cigarette smoke exposure [90], which provides a potential new target for intervention in alleviation of deleterious effects of e-cigarettes.

## Conclusions

Our findings point to significant changes of proteome profiles in V79 cells induced by e-cigarette liquids used at non-cytotoxic, non-mutagenic and non-genotoxic concentrations. Although we used e-cigarette liquid, major findings of this study are in agreement with findings of studies performed using e-cigarette aerosols, indicating that e-cigarette liquid can be used for preliminary screenings when using e-cigarette aerosol is technically unavailable. Proteome analysis revealed reduction in global protein synthesis and impairment of mitochondrial function. The focal adhesion aspect was upregulated which was in agreement with negative impact of e-cigarette liquids on metabolic cooperation. Differences in effect between ECL, ECL-N and nicotine, could indicate the presence of synergistic actions of different components with nicotine. Significant changes in PTMs, often overlooked in standard proteomic analysis, were also detected and need to be addressed in detail in future studies. To appropriately evaluate all the possible health effects of e-cigarettes, providing the complete information on their composition is vital. Our results underline the necessity for further and detailed investigation of potential long-term effects of e-cigarettes.

## Supplementary Information

**Additional file 1: Figure S1. **Effects of e-cigarette liquids on cell viability in V79 cells, as determined by MTT assay (72 h treatment). Data shown as % of control and are the mean ± SEM of three independent replicates. (*p < 0.05; ****p < 0.0001 versus negative control).

**Additional file 2: Figure S2.** Overview of all protein mass spectrometry summary data done in PEAKS PTM engine with two unique peptides and false discovery rate less than 0.1% as protein filters. MS1, MS/MS and chimera are numbers of parent, daughter and chimera spectra, respectively. PSM, peptide spectrum match.

**Additional file 3: Figure S3. **Total ion current profiles of control and treatments nano liquid chromatography runs.

**Additional file 4: Figure S4. **Comparison of unique proteins involvement in biological processess significantly enriched (at least p<0,05 with hypergeometric post-test) among the V79 cell treatments. Graphics and statistics done in FunRich 3.1.3 software version.

**Additional file 5: Figure S5. **Comparison of gene ontologies in molecular function aspect of upregulated proteins. Many of them that are significantly enriched (at least p<0,05 with hypergeometric post-test) among the V79 cell treatments. Graphics and statistics done in FunRich 3.1.3 software version.

**Additional file 6.: Figure S6. **Comparison of gene ontologies in biological processess (BP) aspect of upregulated proteins. Many of them are significantly enriched (at least p<0,05 with hypergeometric post-test) among of V79 cell treatments. Graphics and statistics done in FunRich 3.1.3 software version.

**Additional file 7: Figure S7. **Western blot of Cx43 and tubulin as endogenous control in V79 cells after 72h treatments. Control (1; 5; 9) ECL-N (2; 6; 10) ECL (3; 7; 11) NIC (4; 8; 12).

**Additional file 8: Table S1. **The list of all proteins found in all V79 cell treatments.

**Additional file 9: Table S2. **The list of significantly upregulated and downregulated proteins in V79 cell treatments by protein mass spectrometry relative label-free quantification.

**Additional file 10: Table S3 **Enrichment analysis – cellular component comparison.

**Additional file 11: Table S4. **List of mitochondrial proteins identified uniquely in untreated V79 cells.

**Additional file 12: Table S5. **The list of all PTMs, specific peptides and proteins affected by e-cigarette treatment.

## Data Availability

The datasets generated during and/or analysed during the current study are available in the ProteomeXchange Consortium via the PRIDE partner repository, http://proteomecentral.proteomexchange.org/cgi/GetDataset?ID=PXD032071.

## Abbreviations

e-cigarettes: Electronic cigarettes
ECL-N: E-cigarette liquid with nicotine
ECL: E-cigarette liquid without nicotine
COPD: Chronic obstructive pulmonary disease
SARS-CoV-2: Severe acute respiratory syndrome coronavirus 2
NIC: Nicotine
PASEF: Parallel accumulation serial fragmentation
MTT: Thiazolyl Blue Tetrazolium Blue
DMSO: Dimethyl sulfoxide
HPRT: Hypoxanthine–guanine-phosphoribosyl-transferase
6-TG: 6-Thioguanine
6-TG-R: 6-Thioguanine resistant
PTMs: Post-translational modifications
LFQ: Label free quantification
DDA-PASEF: Data dependent acquisition-parallel accumulation serial fragmentation
PMA: Phorbol 12-myristate 13-acetate
Cx43: Connexin 43
nLC-MS/MS: Nano Liquid Chromatography coupled to tandem mass spectrometry
FAK-1: Focal adhesion kinase 1
COP9: Constitutive photomorphogenesis 9
AGEs: Advanced glycation end products
VPS35: Vacuolar protein sorting-associated protein 35
TNTs: Tunnelling nanotubes
4-HNE: 4-Hydroxynonenal
Met: Methionine
Thr: Threonine
