# Supplementary figures and images for: Electronic cigarette liquids impair metabolic cooperation and alter proteomic profiles in V79 cells
Source: Respir Res. 2022 Jul 15;23:191. doi: 10.1186/s12931-022-02102-w (PMC9285873; doi:10.1186/s12931-022-02102-w)

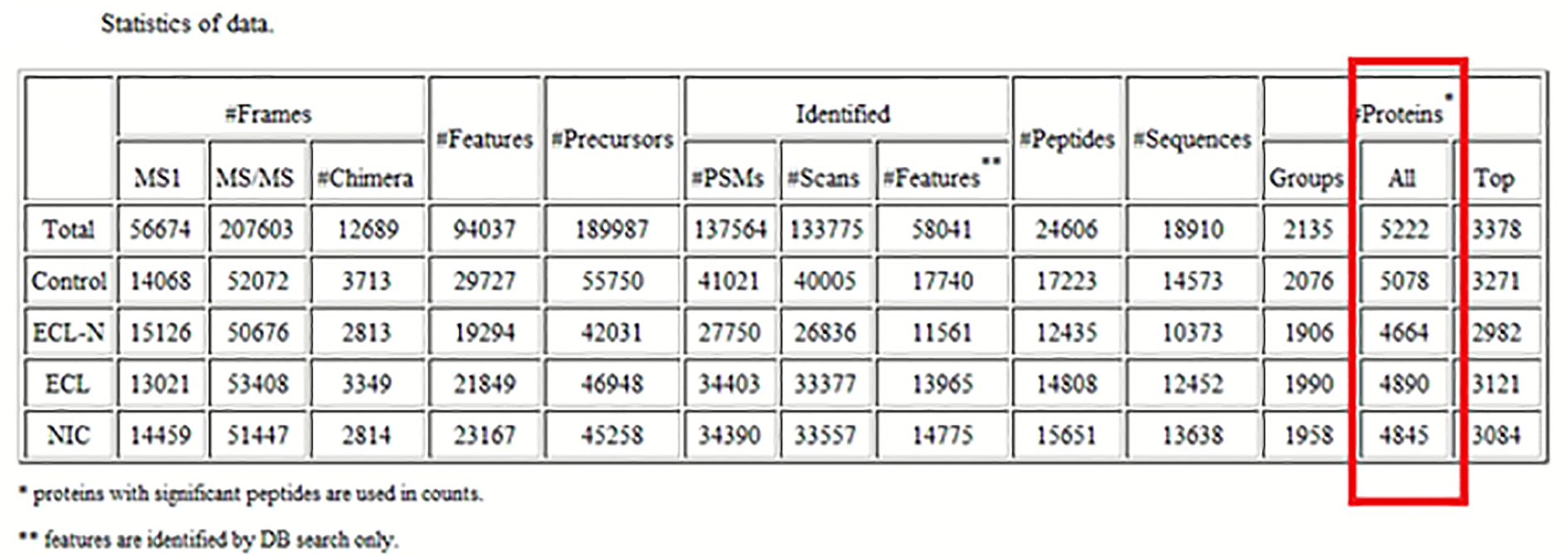

Supplement: Supplementary file 2 — Additional file 2: Figure S2. Overview of all protein mass spectrometry summary data done in PEAKS PTM engine with two unique peptides and false discovery rate less than 0.1% as protein filters. MS1, MS/MS and chimera are numbers of parent, daughter and chimera spectra, respectively. PSM, peptide spectrum match. [file 12931_2022_2102_MOESM2_ESM.tif]

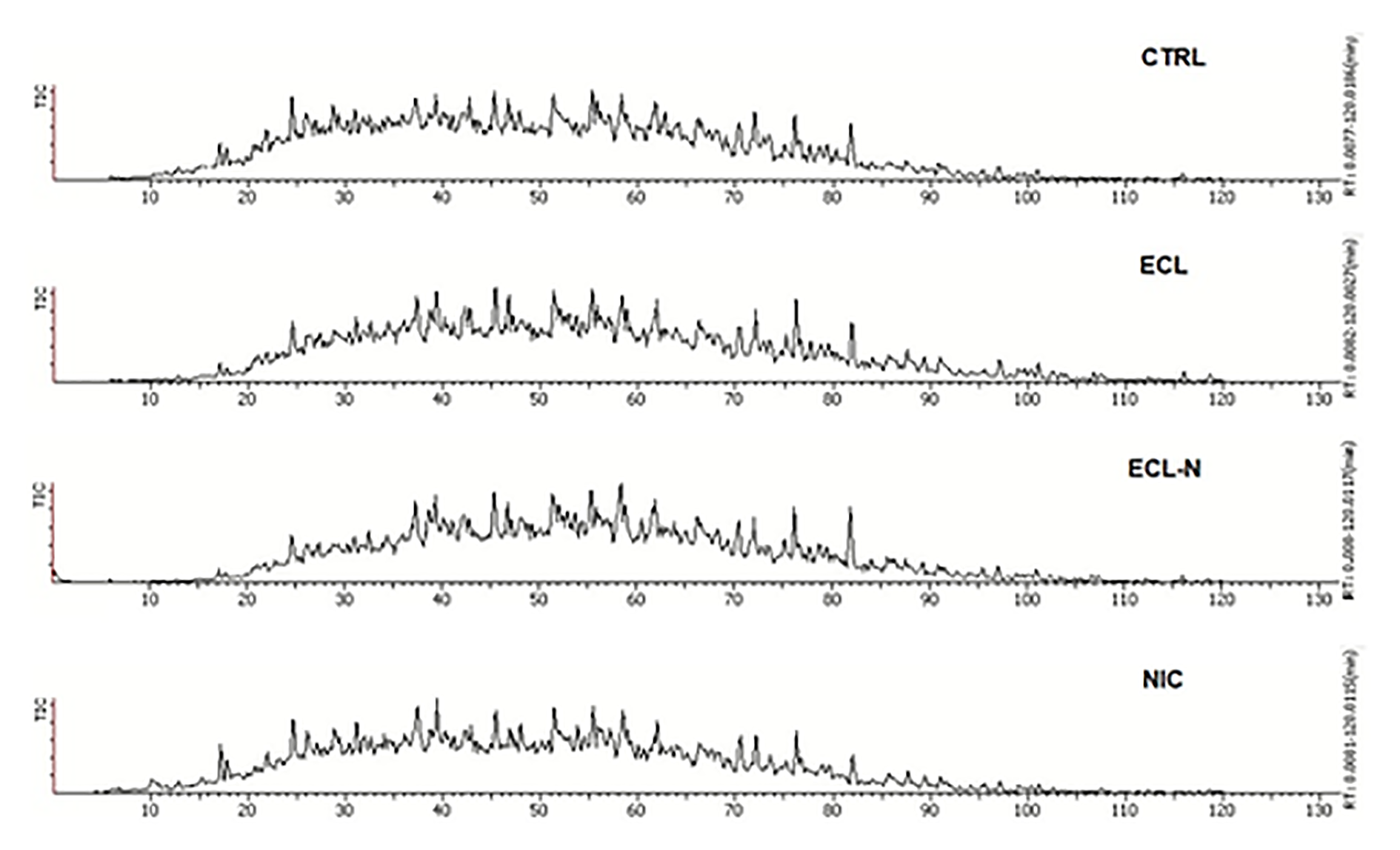

Supplement: Supplementary file 3 — Additional file 3: Figure S3. Total ion current profiles of control and treatments nano liquid chromatography runs. [file 12931_2022_2102_MOESM3_ESM.tif]

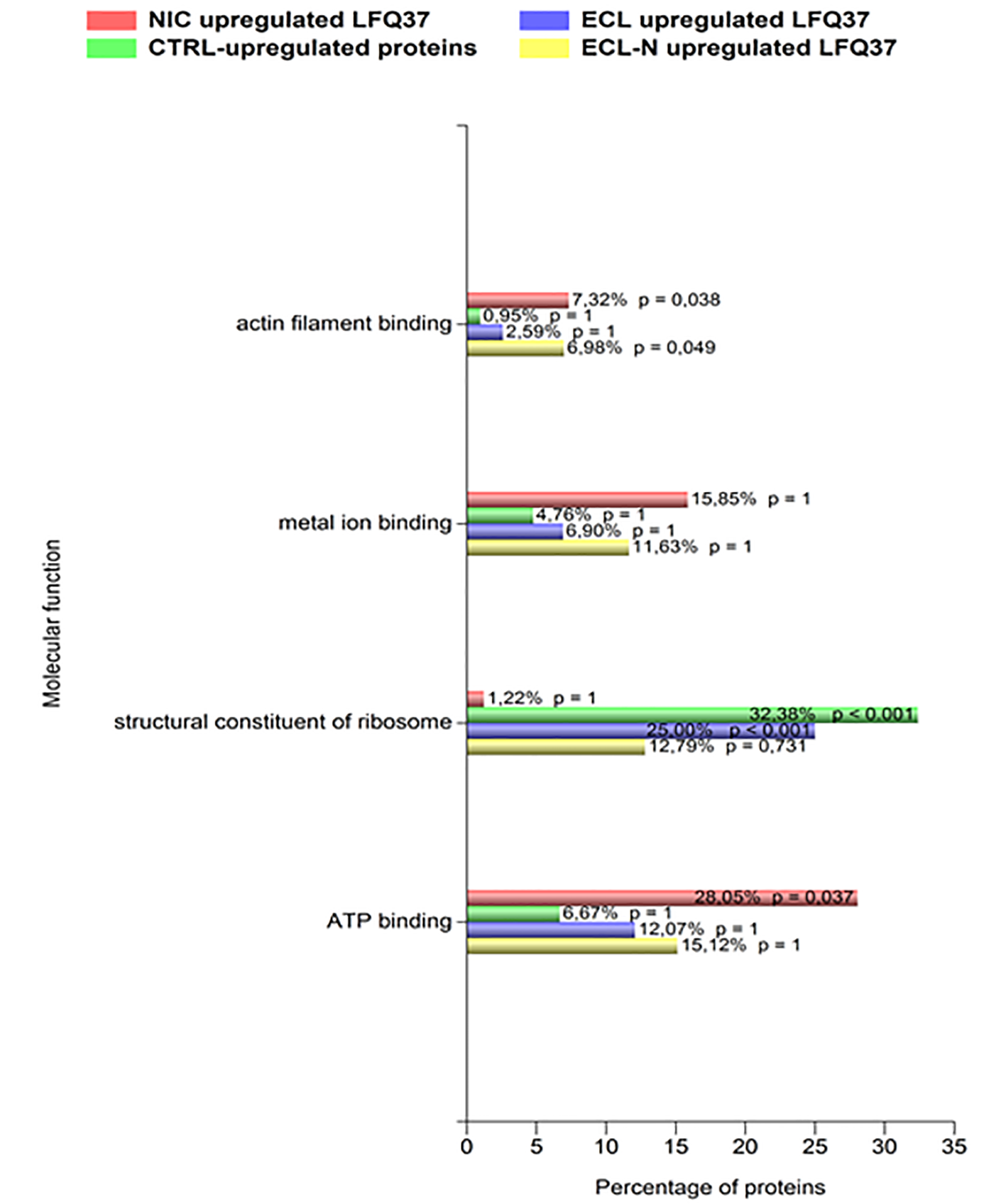

Supplement: Supplementary file 4 — Additional file 4: Figure S4. Comparison of unique proteins involvement in biological processess significantly enriched (at least p<0,05 with hypergeometric post-test) among the V79 cell treatments. Graphics and statistics done in FunRich 3.1.3 software version. [file 12931_2022_2102_MOESM4_ESM.tif]

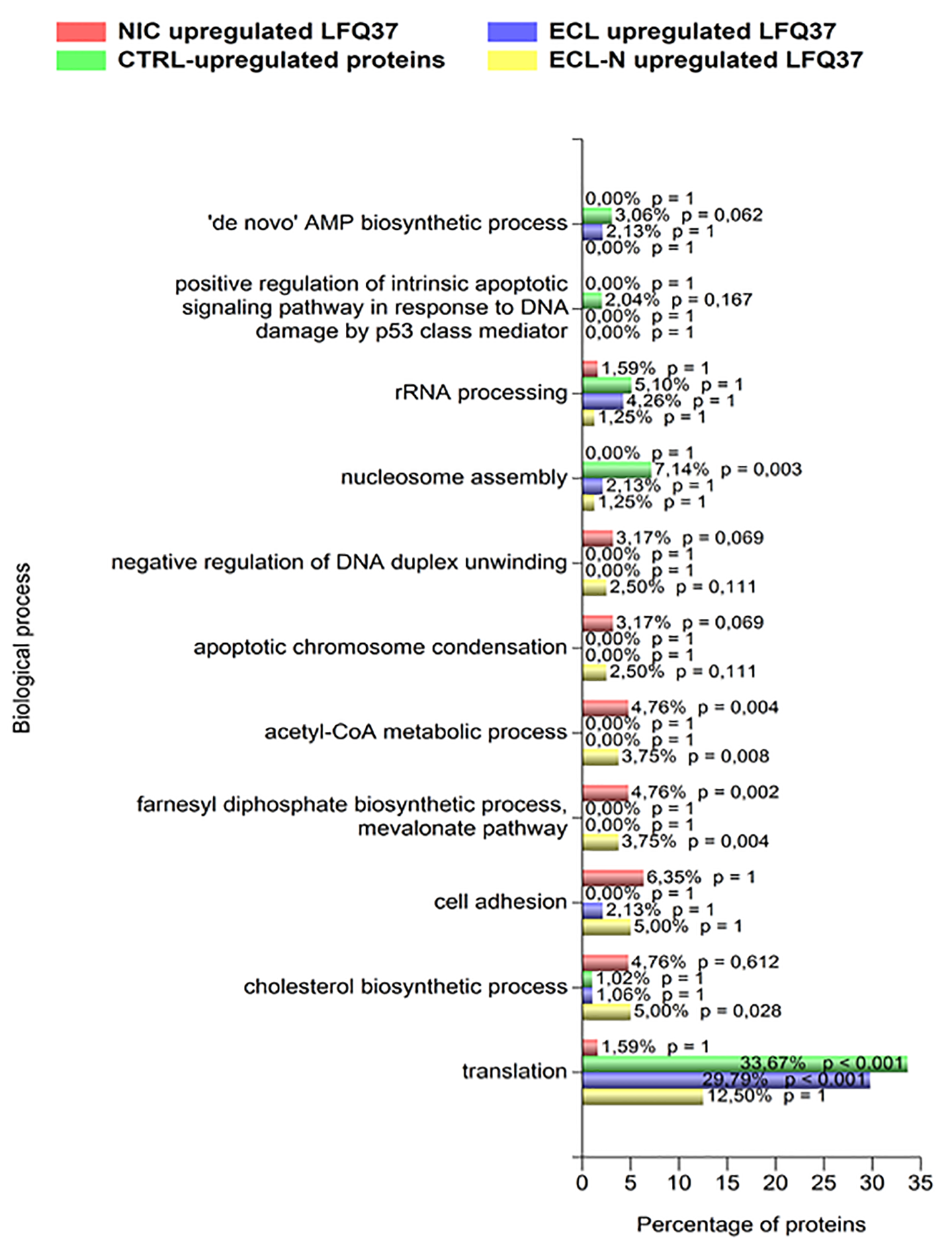

Supplement: Supplementary file 5 — Additional file 5: Figure S5. Comparison of gene ontologies in molecular function aspect of upregulated proteins. Many of them that are significantly enriched (at least p<0,05 with hypergeometric post-test) among the V79 cell treatments. Graphics and statistics done in FunRich 3.1.3 software version. [file 12931_2022_2102_MOESM5_ESM.tif]

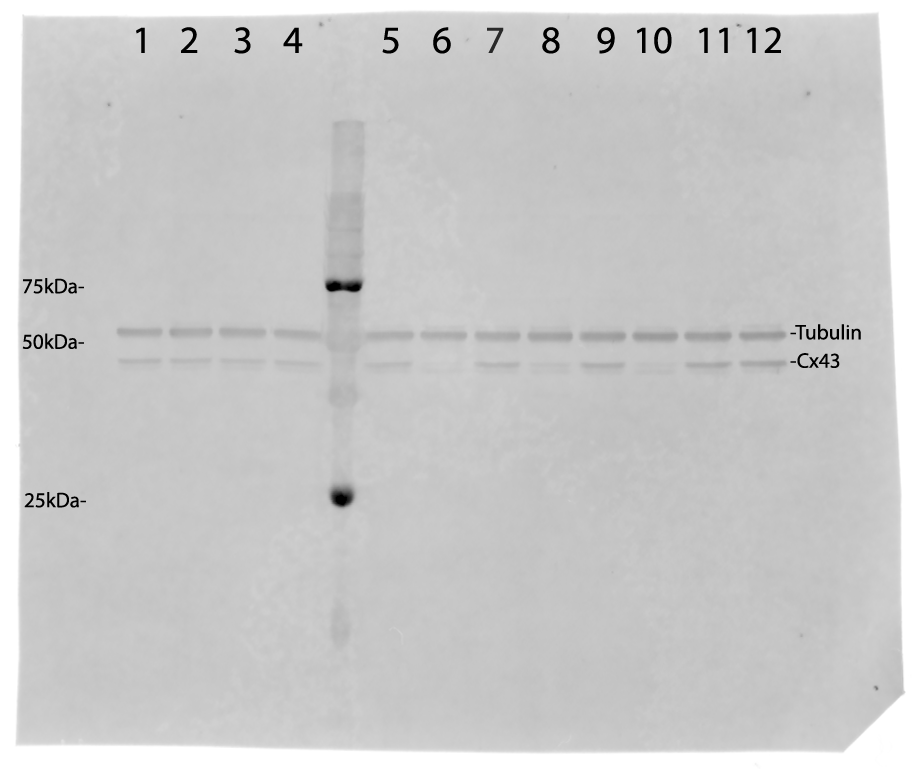

Supplement: Supplementary file 6 — Additional file 6.: Figure S6. Comparison of gene ontologies in biological processess (BP) aspect of upregulated proteins. Many of them are significantly enriched (at least p<0,05 with hypergeometric post-test) among of V79 cell treatments. Graphics and statistics done in FunRich 3.1.3 software version. [file 12931_2022_2102_MOESM6_ESM.tif]
